# Postprandial Glucose Response in Type 2 Diabetes Mellitus Patients and Possible Antioxidant Properties of a Plant-Based Snack Bar

**DOI:** 10.3390/foods13244123

**Published:** 2024-12-20

**Authors:** Maria Dimopoulou, Alexandra Bargiota, Eleftheria Barmpa, Zozo Outskouni, Dimitrios Stagos, Varvara Trachana, Odysseas Androutsos, Olga Gortzi

**Affiliations:** 1 Department of Agriculture Crop Production and Rural Environment, School of Agriculture Sciences, University of Thessaly, 38446 Volos, Greece; mdimopoulou@uth.gr; 2 Department of Endocrinology and Metabolic Diseases, Faculty of Medicine, School of Health Sciences, University Hospital of Larissa, University of Thessaly, 41334 Larissa, Greece; mparmpa.el@gmail.com; 3 Department of Biology, Faculty of Medicine, University of Thessaly, Biopolis, 41500 Larissa, Greece; zooutskouni@uth.gr (Z.O.); vtrachana@med.uth.gr (V.T.); 4 Department of Biochemistry and Biotechnology, School of Health Sciences, University of Thessaly, Biopolis, 41500 Larissa, Greece; stagkos@med.uth.gr; 5 Laboratory of Clinical Nutrition and Dietetics, Department of Nutrition and Dietetics, University of Thessaly, 42132 Trikala, Greece; oandroutsos@uth.gr

**Keywords:** diabetes mellitus, snack bar, postprandial glucose, antioxidant

## Abstract

Daily, more and more people consume snack bars that may have an impact on blood glucose levels. The aim of the present study was to compare the acute effects of a common snack and a plant-based snack bar (PB) that was developed at the University of Thessaly as a functional diabetic snack on blood glucose and insulin in patients with type 2 diabetes mellitus (T2DM). Adults with T2DM (*n* = 10) treated with oral medications were studied in a randomized, crossover clinical trial. On each trial day, postprandial glucose and insulin levels were measured at 30, 60, 90, and 120 min, and a morning snack containing 25 g of carbohydrate was consumed. The procedure was carried out on 2 days, with one of the test meals being consumed on each day. Consumption of a PB snack bar resulted in a smaller and steeper increase in postprandial glucose and insulin levels compared with the usual snack, and there were significant differences 60 and 90 min after consumption of the two tested snacks. The PB snack bar is rich in protein, fiber, vitamins, and minerals and can therefore be suggested as a nutritious and convenient snack for patients with T2DM. In addition, the extract of the snack bar was tested for its bioactivity in human cell cultures.

## 1. Introduction

Diabesity is the most common term used not only to describe the association of diabetes mellitus with obesity but also to define the new epidemic that is closely linked to physical inactivity and other factors, such as genetics [1]. Medical nutrition therapy includes education on dietary habits that affect a healthy weight and control of blood glucose levels [2] and risk factors [3]. One of the glycemic targets set by the American Diabetes Association (ADA) [2] is the desirable glycated hemoglobin (HbA1c) < 7%, and the European Food Safety Authority (EFSA) based on HbA1c changes in order to give health claims for a new diabetic food product [4].

The strategies for improving postprandial glucose are, except for the medication, lifestyle changes emphasized on nutrition-related [5] and technology-related [6]. As for diet, carbohydrates have the greatest impact on blood glucose levels [7]. Controlling carbohydrate intake is essential [8] in order to maintain blood glucose concentration and prevent hyperglycemia (>250 mg/dL) or other delirious secondary metabolic effects [9]. Carbohydrate counting is a preferred strategy to control blood glucose levels regardless of diabetes type and treatment regimen [5]. The benefits of carbohydrate counting include greater flexibility in meal choices and carbohydrate intake [10] and better glycemic control, particularly in adults [11]. Labeling foods with carbohydrates and consuming a fixed amount of carbohydrates in fixed meals is more common in people with diabetes mellitus who use a basal-bolus insulin regimen [5,12]. Nutrition education remains important, not only for carbohydrate counting but also for tailoring diets to improve diabetes control and overall health [3]. Health literacy should help patients with diabetes mellitus with carbohydrate counting, carbohydrate replacement lists, and nutrition information on food packaging [10]. Meal consumption affects postprandial blood glucose [13]. Simplified qualitative meal size estimated, and a snack has <30 g carbohydrate content [14]. Increasing the amount of protein quantity in a meal leads to dose-dependent effects on postprandial glucose levels in participants with diabetes mellitus [15]. A morning snack is a valuable alternative for individuals with diabetes mellitus for whom accurate carbohydrate counting is a challenge [16].

Diabetic snack bars are formulated to either prevent hypoglycemia or reduce postprandial hyperglycemia [9]. In addition, those who ate lower quality snacks reported feeling hungrier than those who ate higher quality snacks (nuts, etc.) [17]. The low-quality snack group also had poorer cardiometabolic biomarkers (higher lipid levels and insulin resistance) [18]. Evening snackers (especially those snacking after 9 pm) had higher blood glucose levels and worse blood lipid levels after eating, but this effect was reduced when participants ate higher quality snacks [19]. Ultimately, there is no right time to eat a snack, as the quality [20] and the portion [21] are more important, even if the snack is eaten at night [22,23] or in the morning/afternoon [24]. However, the consumption of small snacks, such as snack bars, as a substitute for the morning snack and as an afternoon snack improves both blood glucose control and dietary behavior in the context of diabetes management [24].

In addition, there are new types of snack bars that are promising with regard to diabetes mellitus due to their ingredients [25]. In the search for a natural way to support metabolism and glycemic control in patients with type 2 diabetes mellitus (T2DM), researchers suggest that ingredients such as uncooked starch [22], soy [26], ganyong (Canna edulis), kelor (Moringa oliefera) [27], milk chocolate [28], chokeberry [29], mushrooms [25,30], fruit granola [23], and whole grains [31] could be used as alternative snacks. In fact, previous intervention studies showed an improvement in blood glucose levels and cardiovascular risk markers both after three months of consumption of soy protein snack bars [32] and after two months of daily consumption of snack bars containing 37% chokeberry [29]. The nutrient composition of the test products with aronia was 75 g/100 g carbohydrates, 15 g/100 g fat, 4.2 g/100 g protein [29], and 30 g/100 g plant-based protein powder [32]. Many studies aim to investigate the relationship between the consumption of functional foods as meal replacements and markers of glycemic control [33,34,35,36]. Since meal replacement [24,34], especially the morning snack [37], has shown very good results in dietary interventions in diabetic patients [34,35,36,38], on biochemical markers, this proposed intervention protocol was chosen. The aim of this study was to evaluate not only the glycemic response of a PB snack bar enriched with *Coprinus comatus* powder, which was developed at the Laboratory of Food Technology, Quality Control and Food Safety of the University of Thessaly, in adults with T2DM compared to an isocarbohydrate snack regularly used in the diet of diabetic patients but also its possible antioxidant properties.

## 2. Patients and Methods

### 2.1. Examination of Pb Extract’s Antioxidant Activity in Human Mesenchymal Stem Cells (MSCs)

#### 2.1.1. Preparation of Pb Extract from Snack Bars

The extracts were prepared by mixing 25 g of crushed PB snack bar samples with 100 mL of 70% ethanol in a high-speed homogenizer at 200 W for 30 s. Subsequently, the bioactive compounds were extracted at room temperature for 20 h in a shaking incubator at 200 rpm. The extract was passed through a fine mesh filter (Whatman No. 2, Whatman Int., Ltd., Maidstone, UK), and the supernatant was collected. The resulting extracts were concentrated (to remove methanol) using a rotary evaporator and then freeze-dried. The extracts were kept at −80 °C for the trypan blue exclusion assay and cell treatment.

#### 2.1.2. Cell Culture Conditions

Human mesenchymal stem cells (MSCs) were obtained from the Wharton Jelly of umbilical cords from term gestation newborns after birth, having obtained consent from the parents, as previously described [39]. Isolated MSCs were cultured, as reported previously [40].

#### 2.1.3. Trypan Blue Exclusion Assay

The antioxidant activity of the PB extract in MSCs was examined using non-cytotoxic concentrations. The trypan blue exclusion assay was used to determine cell viability after PB extract treatment. In particular, approximately 200,000 cells were seeded in 6-well plates and treated for 24 h with different concentrations of PB extract diluted in DMEM medium. Cells without treatment were used as a control. After treatment, the cells were detached by trypsinization, centrifuged, and resuspended in PBS. Then, dilution of the cells in a 1:1 ratio with 0.4% trypan blue dye was performed, and the number of viable cells was determined using a Neubauer counting chamber. The number of viable cells divided by the total number of cells was used to calculate the viability of the cells. All experiments were carried out on three separate occasions.

#### 2.1.4. Cell Treatment with Pb Extract

For assessing the PB extract’s effect on MSCs’ redox status, cells were seeded into 75 cm^2^ flasks in DMEM containing 10% FBS. After 24 h incubation at 37 °C in 5% CO_2_, the cells were treated with different concentrations of PB extract in DMEM and 10% FBS and again incubated for 24 h. Then, cells were detached by trypsinization and used for the determination of lipid peroxidation, protein oxidation, and reduced glutathione (GSH) levels.

#### 2.1.5. Assessment of Thiobarbituric Acid Reactive Substances (TBARSs), Protein Carbonyl (CARB), and GSH Levels in MSCs

After treatment with PB extract, the cells were detached using trypsin, mixed with a PBS buffer, and disrupted by vigorous vortexing. The protein concentration in the resulting cell lysates was determined using the Bradford assay. Next, a slightly modified TBARS assay, as described previously [41], was employed. For the assessment of protein oxidation, CARB levels were assessed, as described previously [42]. GSH levels were determined, as described previously [41].

### 2.2. Study Design and Procedure

Individuals with a diagnosis of T2DM, not insulin dependent, aged 30–70 years, clinically and biochemically stable, without any acute metabolic complications of diabetes, were also considered for this study. Statistical analyses were performed on the data of 10 subjects (5 males and 5 females) who completed this study. These numbers were chosen based on the literature where similar numbers had provided adequate power [26,38,43,44]. We chose this study design, in which each participant received all interventions and acted as his/her own control, to maximize statistical power and to allow comparison of data at both the individual and group levels. An informed written consent was obtained from the participants. All procedures followed were conducted according to the guidelines laid down in the Declaration of Helsinki and following ethical approval provided by the Bioethics Committee of University of Thessaly, Volos (Approval no. 72/10 July 2023) and registration by the Council of University Hospital of Larissa (Approval no. 8882/28 February 2023).

The participants were selected by convenience purposive sampling from the diabetes patients who were under medical care at the Endocrinology and Metabolic Diseases clinic of the University Hospital of Larissa. The criteria of T2DM diagnosis were the inclusion criteria, and metabolic disorders, obesity, gastrointestinal disorders, and other medical conditions that affect glycemic control were the exclusion criteria [45].

**Figure 1:**
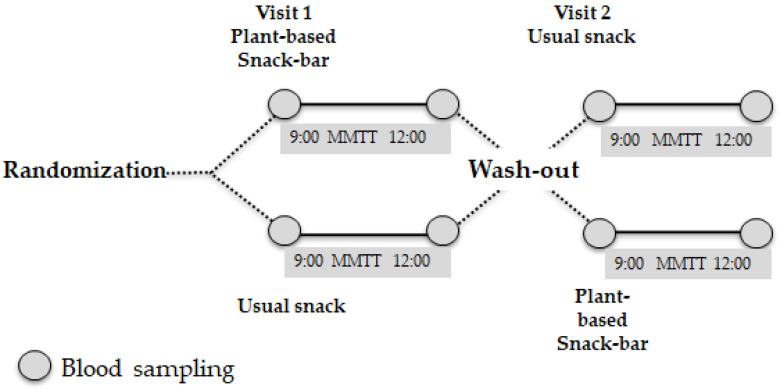
Study design and procedure: the participants underwent the mixed meal tolerance test on 2 separate days, 1 week apart. The participants were randomly assigned to two groups and had a plant-based, mushroom powder-fortified snack bar (PBSB) or a usual snack. After 1 week, the participants were provided with the PBSB and the usual snack in the reverse order. MMTT: mixed meal tolerance test, blood samples: glucose, insulin, etc.

The study procedure took place from November 2023 and was completed before May 2024. At the Metabolic Unit of University Hospital of Larissa, participants had four face-to-face contacts with the medical provider and mainly the investigator, which included (in the order of occurrence):

- Visits 1: consuming 50 g of glucose dissolved in 300 mL of water, which served as the standard food (screening period).
- Visits 2: before the two meal tests, an interview was performed to obtain information on the participants’ demographics, diabetes duration, history of diseases, medications, and the dose and the schedule of hypoglycemic agents. The participants were asked not to change their diet and physical activity during this study. Additionally, they answered a 24 h dietary recall for energy intake assessment and completed a Food Frequency Questionnaire (FFQ) [46] and the International Physical Activity Questionnaire (IPAQ) [47] to evaluate their lifestyle habits.
- Visit 3 and 4: Figure 1 represents exactly the steps of the two meal tests with one week between the two meal tests as a washout period. In the second and third weeks, the study participants were provided with and consumed the two snacks, respectively, each portion of which provided 25 g of carbohydrates. When the meal test with the PB snack bar took place, the participants answered an organoleptic acceptance questionnaire [48] to evaluate color, texture, taste, overall liking, and also their satiety assessed by using a visual analogue scale (VAS), adopted from Flint et al. [49], and an adverse effects questionnaire was also assessed during the supervision period.

#### 2.2.1. Blood Collection Procedure

Subjects were asked to attend the Endocrinology and Metabolic Diseases clinic of the University Hospital of Larissa over two days (each day with one type of test meal), which were 1 week apart, during morning hours (8:00 to 8:30 am). Prior to blood sampling and the consumption of the test meals, they remained fasted over 12 h, were restricted to a maximum of 10 min of mild walking, and were advised to refrain from taking their medications during the morning of each measurement. An intravenous cannula was placed in a forearm vein, and blood samples were drawn at a fasting state (T0) and then at 30, 60, 90, and 120 min after the consumption of test meals. At each time point, blood glucose and insulin levels were assessed.

#### 2.2.2. Test Meals

**Figure 2:**
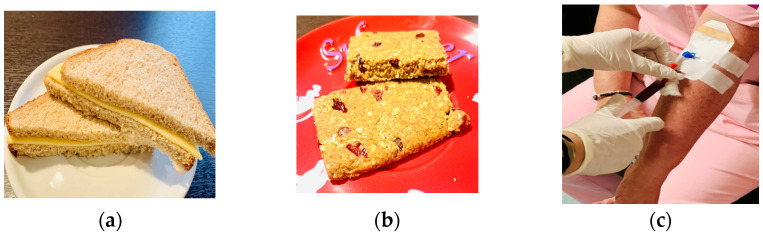
(**a**) Picture of the first snack of this study: 60 g whole grain bread and 25 g low-fat cheese. (**b**) Picture of the second snack of this study: 63 g of PB snack bar. (**c**) Blood collection procedure.

The current study examined the glycemic and insulinemic responses observed after the consumption of two snacks, specifically whole grain bread and low-fat cheese (meal 1) and a plant-based snack bar using mushroom (*Coprinus comatus*) powder (meal 2) (Figure 2). The portion size in the case of each one of the two test meals was adjusted to contain 25 g of carbohydrates. The caloric and nutrient content of the two test snacks is summarized in Table 1. The ingredients of the PB snack bar were the following: rice protein, agave syrup, carob honey, oat bran, oat flakes, lemon juice and zest, orange juice and zest, *Coprinus comatus* powder, almonds, flaxseed, sunflower oil, cranberries, apples, and cinnamon, and also a chocolate coating with stevia that helps lower and control blood sugar, and its development and physicochemical properties have been published in the journal Foods last year [30]. Polyphenols and flavonoids of PB snack bars are due to their ingredients, such as fruits and almonds, omega-3 fatty acids from sources such as nuts and sunflower oil, and psilocybin of the mushroom, which were the main bioactive compounds of the PB snack bars [30] that were produced at a pilot scale (ISO 20613/2019) for the meal tests [30].

#### 2.2.3. Anthropometric Measurements

Anthropometry was carried out at each visit, and patients participated in the same anthropometric measurements step by step, and the same materials (weighing scale, measuring inflexible bars, and blood monitor) were used with the study of Gortzi et al. [3].

#### 2.2.4. Biochemical Indices

All the laboratory investigation took place at the Department of Endocrinology and Metabolic Diseases, and biomarkers were estimated according to the methodology that has been described in the previous study (2024) of the same team [3] and the previous study by Tsirona et al. [50]. As concern trace element levels were determined using the wet acid digestion method that has been described by Wolide et al. [51]. Levels of vitamins have been described by Kostoglou-Athanassiou et al. [52] and Raizada et al. [53]. A step-by-step description of study’s procedures has been contained in Supplementary Materials Section.

### 2.3. Statistical Analysis

All data are reported as mean (standard deviation: s.d.) and as mean change (95% confidence interval: C.I.) from T0. The Kolmogorov–Smirnov test was used to examine the normality of the distribution of the examined variables. Repeated measures analysis of variance (ANOVA) was used to evaluate the significance of the differences between groups at T0, T30, T60, T90, and T120 (treatment effect), the significance of the changes observed within each group (time effect), and the treatment × time interaction effect. Adjustments were made for certain potential confounders (i.e., gender, age, medication, and BMI). The between-group factor was the study groups (i.e., postprandial glucose levels or each one of the two test meals); the within-group factor was the time point of measurement (i.e., T0, T30, T60, T90, and T120). Statistical analysis was conducted with the SPSS statistical software for Windows (version 21.0). The level of statistical significance was set at *p* ≤ 0.05.

## 3. Results

### 3.1. Cell Viability Assay

**Figure 3:**
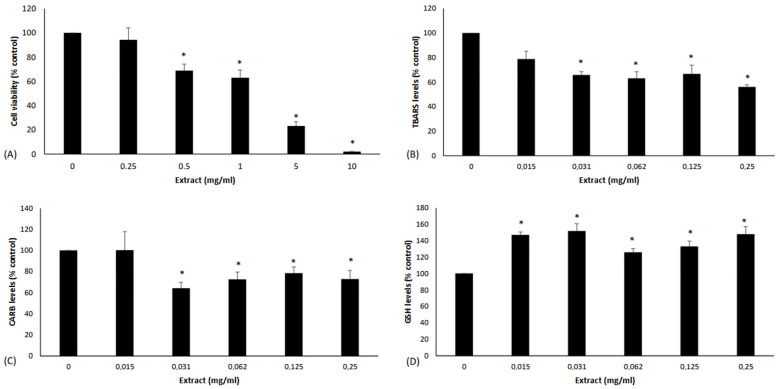
(**A**) Cell viability following the treatment of MSCs with PB extract. (**B**–**D**) Effects of HME on TBARS, CARB, and GSH levels in MSCs after PB extract treatment. All values are presented as the mean ± SEM of 3 independent experiments. * Statistically significant compared to control (*p* < 0.05).

For examining PB extract’s effect on the redox status of MSCs, non-cytotoxic concentrations were selected according to the results of the trypan blue cell viability assay. The results of the trypan blue assay showed that PB extract decreased MSCs’ viability significantly at concentrations higher than 0.25 mg/mL (Figure 3A). Thus, non-cytotoxic concentrations, ranging from 0.015 to 0.25 mg/mL, were used for all the following assays.

### 3.2. Effects of PB Extract on Oxidative Stress Markers in MSCs

The results showed that PB extract treatment of MSCs decreased TBARS significantly by 34.2, 36.91, 33.3, and 43.91 at 0.031, 0.062, 0.125, and 0.25 mg/mL, respectively, compared to untreated cells (Figure 3B). Furthermore, PB extract treatment significantly reduced CARB levels by 35.89, 27.56, 21.53, and 27.10% at 0.031, 0.062, 0.125, and 0.25 mg/mL, respectively, compared to the control in MSCs (Figure 3C). Finally, GSH levels were increased significantly by 47.11, 51.80, 25.89, 33.12, and 48.00% at 0.015, 0.031, 0.062, 0.125, and 0.25 mg/mL, respectively, in MSCs treated with PB extract compared to control (Figure 3D).

### 3.3. Characteristics of the Participants at the Baseline and Lifestyle 

#### Descriptive Characteristics

Subjects’ baseline anthropometric characteristics and other factors such as physical activity, blood pressure, daily calorie intake, etc., and also biochemical variables are shown in Table 2.

It is hopeful that 3 out of 10 participants were normal weight. BMI and WC were 34.3 ± 5.4 kg/m^2^ and 108.1 ± 22.9 cm [54] or 28.8 ± 5.0 kg/m^2^ and 107.4 ± 26.0 cm [3], respectively, according to the two last studies (2020 and 2024) of the University of Thessaly in patients with T2DM. The findings of this study are pretty much the same as the last one. Concerning the lipidemic profile of participants, two males and two females had lower HDL-c levels than 45 mg/dL, but the triglyceride levels were elevated in two female participants. HbA1c is about 5.9% in subjects, which may be due to receiving metformin or the diet pattern they follow. HbA1c was 6.96% in a previous study of patients with T2DM in Thessaly (2016) [50]. Fasting plasma glucose for most participants was on an average of 105 mg/dL, which was the target according to the ADA [55]. Finally, low normal TSH levels (0.4–2.5 mU/L) were associated with a higher number of subjects with HbA1c < 7% in a previous study [52]. According to the FFQ answers, whole grain bread with cheese could be characterized as the most common food choice, either due to the fact that it is an on-the-go morning snack so easy to be consumed wherever or due to a higher degree of liking. The dietary preferences of the consumers seem to be the same as in previous studies [56,57]. As far as acceptance of the PB snack bar, according to the answers of the participants, 90% would prefer the tester PB snack bar compared with other snack bars from the market, and the new product had holistic acceptance [48]. All participants tolerated the new PB snack bar well without any gastrointestinal side effects and mentioned satiety and sense of fullness according to the VAS answers [49].

### 3.4. Meal Test

**Figure 4:**
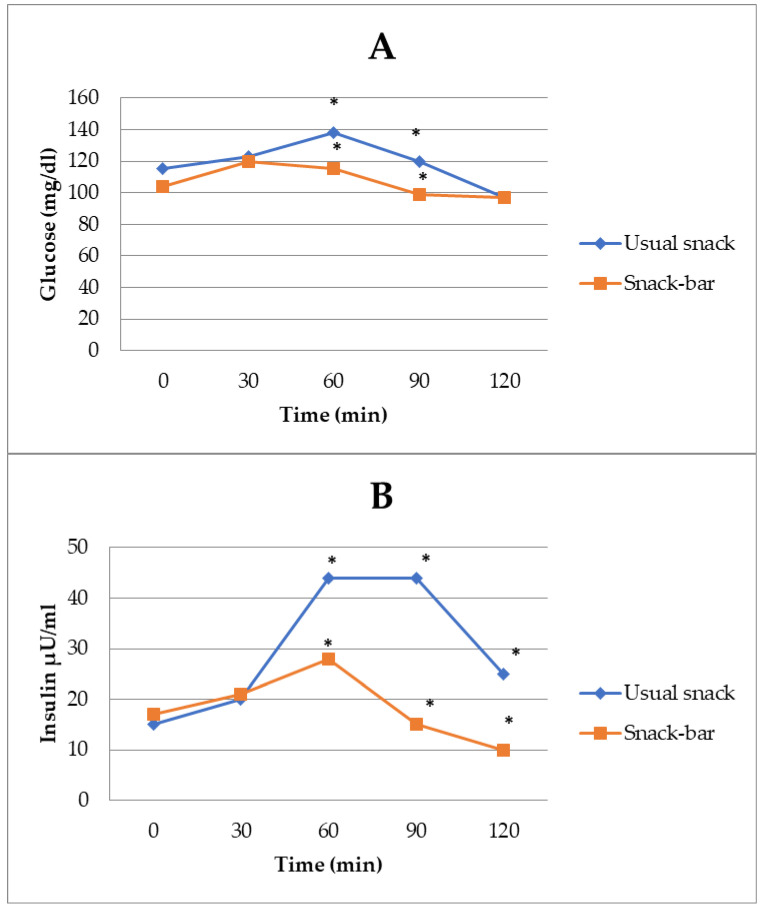
The postprandial glucose (**A**) and insulin (**B**) levels at 2 h. All values were derived from repeated measures ANOVA, using gender, medication, and BMI as covariates. Values with asterisks are significantly different from each other (*p* < 0.05).

The glycemic response to the tested snacks had a gradual rise before reaching its peak at 60 min for both of them (Table 3). The postprandial glucose levels at 2 h were lower compared with the time after consumption (Figure 4A). There was an effect of treatment, time (*p* < 0.05), and treatment × time interaction (*p* < 0.001) on the mean cumulative blood glucose concentration over 120 min. Therefore, blood glucose was reduced overall following the PB snack bar treatment when compared to the usual snack treatment. The analysis of blood glucose at the individuals time points showed that there was an effect of treatment, meaning blood glucose was reduced following the PB snack bar at 90 min and 120 min. Additionally, there was an effect of treatment, time (*p* < 0.05), and treatment × time interaction (*p* < 0.001) on the mean of cumulative insulin concentration over 120 min. Therefore, insulin was greater overall, following the usual snack treatment compared to the PB snack bar treatment (Figure 4B). There was an effect of treatment on insulin at 90 min (*p* = 0.039) and 120 min (*p* = 0.033), indicating that blood insulin was increased at these time points for the usual snack when compared to the PB snack bar treatment.

Use of the PB snack bar compared with a usual snack was associated with a statistically significant decrease in peak of postprandial glucose, incremental glucose response, mean blood glucose level, and glucose variability.

## 4. Discussion

T2DM is one of the most common non-communicable diseases and a global health problem with increasing rates that requires dietary management [55]. Although snacking is an essential component of diabetic management [5], a few clinical trials have investigated the acute metabolic effects of snacking in T2DM patients [31,34,35,38,43,44].

The present study aimed to address this gap in the literature and compared the effects of a novel plant-based snack bar (PB) on postprandial glucose and insulin levels with those after the consumption of a conventional snack (Figure 1), while also evaluating its potential antioxidant properties. First, the PB extract from the bar was tested for its bioactivity in human cell cultures (Figure 3A). Oxidative stress is known to be involved in both the causes and consequences of DM [58]. In particular, a diet high in fat and sugar contributes to the build-up of advanced glycation end products (AGEs), which are formed by the glycosylation of proteins, peptides, and other aminoglycans [59]. Excessive accumulation of AGEs together with their interaction with the receptor RAGE has been shown to increase oxidative stress [58]. Reactive oxygen species (ROS) generated by AGEs-RAGE exacerbate pancreatic β-cell dysfunction and interfere with insulin secretion, leading to blood glucose deregulation and DM [60]. In addition, ROS can cause cytotoxicity to renal cells and promote inflammatory and fibrotic responses in T2DM patients [58]. At the same time, ROS activate signaling pathways such as NF-κB, which increase inflammation and thus exacerbate the complications of DM [61]. Therefore, ROS are increasingly recognized as molecular targets for the prevention of DM and/or the treatment of its vascular complications [58]. Therefore, PB extract has been tested for its antioxidant activity in human umbilical cord MSCs, which are commonly used in DM research [62]. Treatment of MSCs with PB extract improved their redox status. In particular, PB extract increased GSH levels, one of the main antioxidant mechanisms (Figure 3D) [63], in MSCs. Interestingly, activation of the GSH system has been shown to protect against DM-induced renal tubular injury [64]. Moreover, the increase in GSH could explain, at least in part, the ability of the PB extract to reduce protein oxidation (i.e., CARB levels) (Figure 3C) and lipid peroxidation (i.e., TBARS levels) (Figure 3Β). Protection against lipid peroxidation is important in DM. For example, lipid peroxidation caused by hyperglycemia has been reported to weaken the immune system of T2DM patients [65], as it can have deleterious effects on various cellular components, especially cell membranes, ultimately leading to cell death [66]. The decrease in protein oxidation in T2DM is also of importance as it has recently been shown to be a useful marker for monitoring DM complications [67]. It should also be noted that the tested extract exhibited stronger antioxidant activity in MSCs than a functional sports beverage investigated in one of our previous studies [41]. Specifically, the tested extract showed a reduction in TBARS and an increase in GSH at concentrations at least four times lower than the functional sport beverage [41].

The most important finding of this study with the participating T2DM patients was the lower postprandial rise in glucose and insulin after consumption of the PB compared to the usual snack (Figure 4A,B). These results could be due to the different nutritional profiles of the tested snack bar and the usual snack. They differed in the amount of energy of almost 26 kcal, in the protein content of 1 g/serving, and in the ratio of complex and simple sugars (the ratio was almost 1:1 and 2:1 for the usual snack and the PB snack bar, respectively) (Table 1). Soluble fiber reduces postprandial glucose absorption by altering the viscosity of the stomach [68]. Several mechanisms have been described for the effect of soluble fiber, from increasing the viscosity of the chyme to the production of short-chain fatty acids by fermentation, which stimulates gastrointestinal motility and the release of the hormones GLP-1 and PYY [69].

In this study, insulin levels were significantly lower after consumption of the PB snack bar compared to the usual snack, possibly due to the higher ratio of insoluble to soluble fiber, and these results are consistent with a previous study (Table 3) [70]. It should be noted that most participants were diagnosed with T2DM 5 years ago, and all patients were receiving metformin [71].

Another parameter that could explain these findings is the higher protein content of the PB snack bar compared to the usual snack, which could have affected the postprandial glucose response. In a study by the ADA (2002), protein-enriched snack bars were compared with rice protein bars, and it was shown that the intake of protein can play a decisive role in postprandial glucose levels [72]. In addition to the amount of protein, the protein source, which was different in each snack, may have influenced the results. More specifically, the PB snack bar contained mushroom powder (*Coprinus comatus*) as an ingredient. PB protein powders are gaining ground in the diabetic food market [30,73] as they appear to have effects on postprandial glucose peaks. Although this has no bearing on the acute effects of the PB snack bar tested in this study, interestingly, a substance found in mushrooms called psilocybin has been found to prevent the loss of critical insulin-producing cells in the pancreas, which could be an additional positive long-term effect of consuming the PB snack bar [74]. However, evidence is lacking, and further studies should shed more light on this speculation.

The best-studied ingredients for the treatment of T2DM are undoubtedly dietary fibers [75]. Dietary fibers seem to be able to control blood glucose levels, and therefore, in this study, they were the main source of carbohydrates of the PB snack bar. In fact, the PB snack bar had a higher fiber content compared to the regular snack bar (1.8 and 0.96 g/serving, respectively). Barley and oatmeal as natural sources of β-glucan to improve lipid biomarkers other than glucose metabolism were also investigated by Reiners et al. (2023) [76]. In a double-blind, randomized, placebo-controlled study, Gourineri et al. (2020) [77] showed that glucose and insulin concentrations decreased significantly after consuming two snacks containing 21 g and 30 g of fiber compared to a control bar containing 2 g of fiber. In another study, snack bars with a higher fiber content showed favorable satiety results and a low glycemic index (GI) [17].

Another reason that could explain the results related to glucose and insulin response is the higher amount of polyphenols and flavonoids in the PB snack bars due to their ingredients, such as fruits and almonds [30], which can inhibit α-amylase activity, and anthocyanins in particular can inhibit alpha-glucosidase activity and lower postprandial blood glucose levels. Although the mechanism has been described [78], it is not clear at what dose the flavonoids and polyphenols of each product can affect insulin secretion and regulate blood glucose levels. Some researchers studied not only 893–533 mg of anthocyanins per day with unpredictable results [29] and others 33 mg of isoflavones with a significant decrease in hemoglobin A1c (24.19 [7.29] mmol/mol) [32] but also the acute effect of snack bars with 1–2 g of total polyphenols, which had a positive effect on postprandial glycemia [79].

In terms of food labeling, most granola bars contain a small amount of fiber, with one bar containing only a few grams of fiber [22]. Looking at the ingredients, we find that they contain a small amount of oat flakes [30], but the rest of the cereals are rice flour and wheat flour. So most of the fiber comes from artificial sources (it can be considered “natural” in a way, as it is usually extracted from the chicory root, but this requires processing, and such fibers are called functional). In this case, we are talking about inulin and oligofructose, both sources of fiber with prebiotic effects. Research has not confirmed or shown whether they really have the same positive properties as natural fiber from fruits, vegetables, legumes, and whole grains [80,81]. Unfortunately, the product label does not mention where the few grams of fiber per bar come from, but most likely, the fibers are from these artificial sources, as whole grains are scarce [9,17,30,82]. Finally, the fiber in combination with other ingredients contained in these bars may likely have beneficial properties in terms of controlling blood glucose levels and lowering cholesterol [30]. Therefore, it would be helpful if the main claim of these products was for fiber only, and the definition of prebiotics was consistent with the recommendations of the clinical trials [83], although this is not required by EFSA and Food and Drug Administration (FDA) regulations [81]. The EFSA considers a daily intake of more than 25 g of dietary fiber to be sufficient to reduce diabetes complications such as coronary heart disease [84] and to improve weight maintenance, which is associated with an improvement in postprandial glucose levels [85].

However, the consumption of such fibers seems to increase the occurrence of flatulence, nausea, and other gastrointestinal symptoms [86]. The study participants had no side effects after consuming 63 g of PB snack bars (almost 3 bars), and the acrylamide content was <0.1 μg for each snack bar, according to a previous chemical analysis published in 2023 [30], to make the product safe for the participants and below the limits set by the European Commission in 2017 in Regulation 2017/2158/EC [87] for oat, spelt, barley, and rice-based products (150 μg/kg). No side effects were observed even after eating the usual snack. Wholemeal bread also supports intestinal health and reduces inflammation. The acrylamide content of most types of bread is 135 μg/kg [88] and thus below the limit of 300 μg/kg, according to the regulation [87]. Not only the carbohydrate value but also the fat content was investigated, with a focus on unsaturated fatty acids as part of a balanced snack for T2DM patients. Replacing rapidly absorbed carbohydrates with a fat source rich in monounsaturated fatty acids to achieve improved glycemic control in these patients was used as an important adjunctive therapy in a randomized trial of 203 Chinese patients with T2DM [36]. The comparison of two foods (drink versus bar with the same composition) showed that the drink resulted in better control of plasma glucose levels and postprandial insulin levels. In another study [35], in which 15 subjects with T2DM (35–87 years) consumed a soy bar (80 kcal = 30 g) or test cookies (1 pack = 115 g), the blood glucose response in the soy bar test was significantly lower than in the cookie test, and the insulin response was also significantly lower. Although the results differed from those of the present study, the changes paralleled the observed higher or lower glucose and insulin levels [35].

Finally, the snack bars could be an alternative at any time, especially as a convenient food choice as part of a personalized diet plan with potential health benefits. Two recent surveys [34,89] also replaced a meal, specifically breakfast, with granola bars, with the first survey conducted by the American Diabetes Association in 2002 [38]. Higher fiber intake was associated with lower postprandial glucose at breakfast, and soluble fiber intake from foods and supplements had a similar effect in patients with type 2 diabetes [89]. Lower postprandial blood glucose was observed in both patients with T2DM and participants with normal glucose tolerance [34]. Higher fiber intake is a healthy dietary habit of participants [46] and includes especially morning foods such as cereals and toast, which are often preferred by patients with diabetes [56,57]. The influence of foods such as cereal, oatmeal, and toast was investigated by Chang et al. (2019) to prevent postprandial hyperglycemia in T2DM patients and improve glycemic variability [56]. In this study, no significant differences in anthropometric parameters were found (Table 2), but in a previous study, eating a morning snack during the day to align meals with the body’s circadian rhythms was found to accelerate basal metabolic rate and provide more health benefits [18]. Participants in another 12-week weight management program were instructed to eat two meal replacements (190 calories each) plus two snacks of 100 to 200 calories at breakfast, which had a positive effect on weight management [90]. Diabetic snack bars vary in energy content and range from 100 kcal [9] to 131 kcal [17], 143 kcal [82], or even 253 kcal [19]. The PB snack bar provides 101 kcal/portion based on previous diabetic snack bars with similar energy and lower carbohydrate content to improve hyperglycemia and maintain blood glucose concentrations at levels above those that could cause adverse secondary metabolic effects, just like snack products with health claims [9].

The nutrition claim of the novel PB snack bar is “source of protein and dietary fibres” [30]. Except for the nutrition claims, the potential anti-diabetic properties of them could also be a result of their ingredients, such as species (cinnamon), fruits (cranberries), and whole grains (oat, rye, and brown rice) [30], that have been presented [78] in a recent study and their mechanism of action by reduction in HbA1c, AGEs, and inhibiting carbohydrate-hydrolyzing enzymes (decreased a-glycosidase, a-amylase) [78]. As a consequence, many health claims about cereal bars have been proposed, and their functionality was attributed [91] mainly because of their ingredients, either extracts or even powders of by-products [92]. These ingredients emphasized their impact on blood sugar regulation [93] and even the dose-dependent mode of action of fiber intake and plasma glucose change. Overall, there are fewer studies on their use in the treatment of dyslipidemias [94], but there are some studies that highlight their antioxidant properties in vitro [95,96] and ex vivo [97].

So the biggest challenge was the development not only of a PB snack bar with a nutrient profile and carbohydrate value that could contribute to achieving and maintaining near-normal glycemic levels but also with a high antioxidant profile due mainly to the almonds, cranberries, oranges, and lemon juice. Spectroscopic methods were used in a previous study (2023) of the same team to assess the antioxidant activities of the PB snack bars [30]. Researchers found that different amounts of the same bioactive compound could change the peak of glucose concentration 60 min after the ingestion of whole grain snack bars and range from 98.29 mg/dL to 168.36 mg/dL [31]. Christiansen et al. (2023) [79] found a slight improvement in glycoregulatory hormone and glycemic responses to a high-carbohydrate food containing 1.2 g of polyphenols in young adults but did not affect appetite or oxidative stress responses. In another study, pineapple snack bars had better results than cranberry-based snack bars [20], and the glucose levels reduced and were pretty much the same 90 min and 120 min after consumption, which agrees with the results of the tested snack bar of this study. According to Bae et al., cranberry-based snack bars lead the way in terms of glycemic response after the consumption in T2DM patients but not in healthy adults [34]. The patients with T2DM noticed between 60 min and 90 min the higher changes in glucose levels, which ranged from 200 mg/dL to 230 mg/dL. This result is fully in disagreement with the glucose responses of both tested snacks of the University of Thessaly. Another difference between the two studies is that post-meal plasma glucose levels were significantly lower compared with pre-meal levels in the present study compared with the study by Bae et al. [34].

Last but not least, sweeteners such as date syrup [10], manuka honey [17,82], and maltodextrin [38] require further investigation on glucose regulation and their possible anti-diabetic effect, although they have already been used in diabetic snacks. The acceptance daily intake values for non-calorie sweeteners such as acesulfame K, aspartame, saccharin, cyclamate, sucralose, and stevia are 15, 40, 5, 7, 15, and 4 mg/kg/weight, respectively, and the maximum consumption has already been determined [98]. The advantage of the tested PB snack bar was the use of natural products such as date syrup and carob honey that were used as sweeteners [30].

Another highlight of this study is that mushroom powder-fortified snack bars could be an idea for new diabetic food products based on funjies [30,73], as they seem to have an impact on peak postprandial glucose levels [73]. Besides the nutritional profile of the two tested products not being the same, another difference between the two food products is the mushroom powder in the PB snack bar, except for the other no animal-derived ingredients of the snack bar compared with the toast that the main source of protein is cheese (Figure 2). According to preliminary research published in the journal Genes, a compound found in mushrooms named psilocybin might help prevent the loss of critical insulin-producing cells in the pancreas [74].

Also, mushrooms in daily consumption improve hepatic health by reducing serum glutamic-oxaloacetic transaminase reduced from 246 to 61 IU/L and normalizing the aspartate aminotransferase and γ-glutamyltransferase activities [99]. In a recent study (2023), the prevalence of elevated liver enzymes is raised by alanine aminotransferase (28.8%), aspartate aminotransferase (23.7%), and alkaline phosphatase (48.8%) out of 135 T2DM patients [100]. In the same study for good glycemic control, patients had HbA1c < 7%, and the range was from 16.6 to 28.1 IU/L for aspartate aminotransferase and 17.7 to 37.8 IU/L for alanine aminotransferase. Abnormal liver function tests in the first study of T2DM patients (2012) mentioned an increase of about 18% and 15% for alanine aminotransferase and aspartate aminotransferase, respectively, but γ-glutamyltransferase activities were normal [101]. The results of our study do not differ a lot from the most recent results as concerns aspartate aminotransferase (≈21%) and alanine aminotransferase (≈21%) [100], but concerning γ-glutamyltransferase activity levels, there is an agreement with the first study [101]. From the nutrition perspective, this reality necessitates a broad understanding of the foods that are empirically considered to be the most anti-inflammatory and so anti-diabetes due to the vicious cycle between insulin resistance and inflammation in nonalcoholic fatty liver disease (NAFLD) [78].

Another comorbidity that has an impact on the mushroom intake is dyslipidemia [25]. Besides, lipid lowering and cardiovascular risk factor screening are goals according to the report by the ADA and the European Association for the Study of Diabetes (EASD) [4]. Total cholesterol levels, low-density lipoprotein cholesterol, and triglyceride levels for most of the participants ranged from 155.77 ± 33.9 mg/dL, 106.99 ± 27.7 mg/dL, and 122.21 mg/dL, respectively, but triglyceride levels were elevated in a few diabetics, and lower HDL levels were found for four of them. Although many studies examine the lipidemic profile of patients with T2DM [3,79], not many studies examine snack bars in the treatment of dyslipidemias [94].

Using plasma biomarkers of participants, the results agree with the previous studies that found negative correlations of aging, including short-chain fatty acid (SCFA) production, vitamin B12 biosynthesis, and amino acid metabolism. The “metatranscriptomic clock” also suggested that vegetarian people had better metabolic profiles [102]. Although the B12 deficiency was more likely to affect female participants of the study and vegetarians [103,104], many diabetic products and formulas have high amounts of vitamin B12 and other micronutrients [39,105,106], like the PB snack bar of this study. Besides plant-based products, it is not only a healthy choice but also a consumer’s demand for a sustainable future [107,108]. Moreover, some studies correlate vegetarian patterns with better biomarkers in T2DM patients, but the Mediterranean diet is the pattern with the antioxidant and anti-inflammation characterization [109] and the protective role against T2DM complications [110]. In a previous study, levels of ferritin, iron, and calcium in the serum of diabetes were 24.96 ± 18.60 µg/L, 55.95 ± 20.99 µg/dL, and 4.28 ± 1.19 mg/dL, respectively [47], and in another study, levels of Fe were 1.23 ± 0.55 mg/L and Ca were 81.03 ± 10.53 mg/L [111]. The levels of the participants of the study were similar, and diabetic patients were characterized by significantly higher levels compared with prediabetes [112]. Vitamin D3 was also examined due to the fact that patients with type 2 diabetes mellitus (T2DM) appear to be at high risk of osteoporotic fractures [113], and unfortunately two women appeared to have deficiency and one inadequate levels of vitamin D3. Vitamin D3 supplementation of 5500 IU/day and Ca 500 mg/250 mL that was mentioned from a woman of the study helped glycemic control remain within the optimal range [114]. However, factors such as age, dose load, frequency, chemical structure, and solubility affect bioavailability [115,116].

The present study has certain strengths and limitations. One of its strengths is the methodological design (RCT with a cross-over design). However, the inability to measure levels of additional hormones regulating appetite is a limitation of this study. Further research with more participants and daily consumption of the PB snack bar as part of a personalized balanced meal plan is required to evaluate the (long-term) effects of the PB snack bar on patients’ with T2DM metabolism and dietary management.

## 5. Conclusions

In conclusion, snack bars may improve glucose control among patients with T2DM. The PB snack bar, which was used in the present study, is packed with key vitamins and minerals, is a source of protein and dietary fibers, contains a relatively small amount of total carbohydrates (9.5 g per portion), and has a 2:1 ratio of complex to simple carbohydrates, and according to the findings of this study, it may cause a lower and steeper increase in postprandial glucose and insulin, thus possibly making it a nutritious and convenient food choice. Future studies could explore consumers’ acceptance of the PB snack bar and its long-term impact on T2DM patients’ clinical management and nutrition care.

## Figures and Tables

**Table 1:** Nutritional profile of a plant-based snack bar compared with the usual snack.

Nutrition Declaration	Snack 1 Study Snack	Snack 1 (Portion) *	Snack 2 Study Snack	Snack 2 (Portion) *
Total energy, Kcal	240	160.9	266	101.4
Protein, g	13	10	11.8	4.5
Fat, g	6.8	4.4	12.6	4.8
Saturated, g	3.6	3.3	9.9	3.8
Carbohydrate, g	25	12.5	25	9.5
Fiber, g	3.6	1.8	2.5	0.96
Total sugars, g	3	1.5	2.94	1.12
Na, mg	0.93	0.5	37.8	14.4
K, mg	0	0	31.5	12
Foods consumed	Whole grain bread, 60 g, and cheese (low fat), 25 g	Whole grain bread, 30 g, and cheese (low fat), 25 g	PB snack bar 63 g	PB snack bar 24 g

* Snack 1/portion (en, %): vitamin B1 1%, vitamin B12 0%, calcium 28%, iron 2%, folate 6%, potassium 2%, magnesium 2%, phosphorus 23%, and snack 2/portion (en, %): vitamin B12 20%, calcium 15%, iron 8%, folate 6%, potassium 10%, magnesium 25%, and phosphorus 20%.

**Table 2:** Descriptive of somatometric characteristics, 24 h dietary recall, and biochemical variables of the study participants in the total sample (*n* = 10).

Characteristics	Total (*n* = 10)
Age (years)	49 ± 8.9
Duration of T2DM (years)	5 ± 1.2
Patients taking glucose-lowering agents (n)	10
Metformin	6
Metformin + statins	3
Metformin + angiotensin receptor blockers	1
Hypertension (n)	1
Dyslipidemia (n)	3
Nonalcoholic fatty liver disease (n)	2
Systolic blood pressure (mmHg)	130 ± 7
Diastolic blood pressure (mmHg)	80.4 ± 4.1
Low physical activity	4
Moderate physical activity	3
High physical activity	3
**Anthropometrics and body composition indices**	
Body weight, kg	79.7 ± 19.2
Body mass index	27.95 ± 5.75
Waist circumference (cm)	105.4 ± 28
Waist-to-hip ratio	1.15 ± 0.15
Body fat (%)	34.4 ± 9.65
Visceral fat (%)	8 ± 0.9
Muscle mass (%)	62.2 ± 7.1
Daily calorie intake (kcal)	1645.55 ± 276.6
**Laboratory investigations**	
Glycated hemoglobin (%)	5.9 (5.2–6.6)
Fasting blood glucose (mg/dL)	109.5 ± 16.7
Total cholesterol (mg/dL)	155.77 ± 33.9
High-density lipoprotein cholesterol (mg/dL)	50.08 ± 9.22
Atherogenic index	3.11 ± 0.63
Triglycerides (mg/dL)	122.21 (59.8–214.2)
Low-density lipoprotein cholesterol (mg/dL)	106.99 ± 24.61
Aspartate aminotransferase (IU/L)	20.86 (13.6–30.7)
Alanine aminotransferase (IU/L)	21.39 (12.6–38)
Alkaline phosphatase (IU/L)	69.6 ± 20.5
γ-glutamyltransferase (IU/L)	15.2 ± 6.7
Thyroid-stimulating hormone (μ IU/mL)	1.62 ± 0.4
Free thyroxine (ng/dL)	1.29 ± 0.4
Creatinine (mg/dL)	0.74 ± 0.15
Albumin (mg/dL)	4.71 ± 0.20
Ferritin (ng/mL)	110.36 ± 40
Uric acid (mg/dL)	4.66 ± 2.38
Folic acid (ng/mL)	5.91 ± 3
P (mg/dL)	3.23 ± 0.6
Fe (μg/dL)	86.88 ± 40
Ca (mg/dL)	9.63 ± 0.6
Na (mmol/L)	139.2 ± 5
K (mmol/L)	4.57 ± 0.4
Mg (mmol/L)	2 ± 0.5
25 OH vitamin D3 (ng/mL)	29.08 ± 5
Vitamin B12 (pg/mL)	320.89 ± 50

**Table 3:** Changes in serum glucose and insulin levels during the two 2 h meal tests.

	T0 Mean (SD)	T30 Mean (SD)	T60 Mean (SD)	T90 Mean (SD)	T120 Mean (SD)	Changes (T120-T0) Mean (95% Cl)	*p* Value * (Treatment × Time)
Serum glucose (mg/dL)							<0.001
Snack 1 (*n* = 10)	114.8 (18)	123.5 (10.9)	138.1 (23) ^a^	120.34 (23) ^a^	97.2 (20)	17.83 (9.7 to 11.8) **	
Snack 2 (*n* = 10)	104 (6.5)	120.3 (7.4)	114.8 (15) ^b^	99.3 (13) ^b^	97 (10.2)	7 (4 to 10) **	
*p* value (treatment effect) ***	0.360	0.380	**0.005**	**<0.001**	0.130		
Serum insulin (μU/mL)							<0.001
Snack 1 (*n* = 10)	14.07 (8)	20.06 (9)	42.05 (26) ^a^	44.06 (18) ^a^	25.7 (15.7) ^a^	10.23 (6.6 to 12) **	
Snack 2 (*n* = 10)	17.38 (6)	21.75 (20)	27.03 (23) ^b^	14.95 (10) ^b^	10.93 (7.3) ^b^	6.45 (2.5 to 14) **	
*p* value (treatment effect) ***	0.973	0.999	**0.007**	**0.039**	**0.033**		

Snack 1: 60 g whole grain bread and 25 g low-fat cheese (WBLFC); snack 2: plant-based snack bar (PBSB). All *p* values were derived from repeated measures ANCOVA, using gender, medication, and BMI as covariates. Bold font indicates statistically significant *p* values (i.e., *p* < 0.05). ^a^, ^b^ Means in a column sharing the same superscript letter are significantly different from each other, *p* < 0.05. * Treatment × time interaction effect, ** within-group comparisons at T0, T30, T60, T90, and T120 (treatment effect), and *** between-group comparisons at T0, T30, T60, T90, and T120 (treatment effect).

## Data Availability

The original contributions presented in the study are included in the article and Supplementary Materials, further inquiries can be directed to the corresponding author.

## Supplementary Materials

The following supporting information can be downloaded at: https://www.mdpi.com/article/10.3390/foods13244123/s1.
