# Supplementary material for: Postprandial Glucose Response in Type 2 Diabetes Mellitus Patients and Possible Antioxidant Properties of a Plant-Based Snack Bar
Source: Foods. 2024 Dec 20;13(24):4123. doi: 10.3390/foods13244123 (PMC11675813; doi:10.3390/foods13244123)
Supplement: Supplementary file 1 [file foods-13-04123-s001.zip › foods-3339104-supplementary.pdf]

Supplementary and Materials

2. Patients and Methods

2.1. Examination of PB extract's antioxidant activity in human mesenchymal stem cells (MSCs)

2.1.1. Preperation of PB extract from snack bars

The extracts were prepared by mixing 25 g of crushed bar samples with 100 ml of 70% ethanol in a high Speed Homogenizer 200W, for 30 seconds. Subsequently, the bioactive compounds were extracted at room temperature for 20 hours in a shaking incubator at 200 rpm. The extract was passed through a fine mesh filter (Whatman No. 2, Whatman Int., Ltd., Maidstone, UK) and the supernatant was collected.The resulting extracts were concentrated (to remove methanol) using Rotary Evaporator, and then freeze-dried.The extracts were kept at −80 °C for Trypan blue exclusion assay and Cell treatment.

2.1.2. Cell Culture Conditions

Human mesenchymal stem cells (MSCs) were obtained from the Wharton Jelly of umbilical cords from term gestation newborns after birth, having obtained consent from the parents, as previously described [39]. Isolated MSCs were cultured, as reported previously [40], in Dulbecco's modified Eagle's medium (DMEM) high glucose with stable glutamine and sodium pyruvate (BioWest, Miami, FL, USA) plus 10% fetal bovine serum (FBS; Thermo Fisher Scientific, Waltham, MA, USA) and 1% penicillin/streptomycin (Thermo Fisher Scientific, Waltham, MA, USA) at 37°C in a humidified atmosphere of 5% CO_2. Cells were maintained in culture in order to produce three different passages (i.e. p18, p23 and p28) that were used for the experiments. The medium was changed twice a week and cells were passed when 90% confluency was reached.

2.1.3. Trypan blue exclusion assay

The antioxidant activity of the PB extract in MSCs was examined using non-cytotoxic concentrations.The Trypan blue exclusion assay was used to determine cell viability after PB extract treatment. In particular, approximately 200,000 cells were seeded in 6-well plates and treated for 24 hours with different concentrations of PB extract diluted in DMEM medium. Cells without treatment were used as control. After treatment, the cells were detached by trypsinization, centrifuged and resuspended in PBS. Then, dilution of the cells in a 1:1 ratio with 0,4% Trypan blue dye was performed and the number of viable cells was determined using a Neubauer counting chamber. The number of viable cells divided by the total number of cells was used to calculate the viability of the cells. All experiments were carried on three separate occasions.

2.1.4. Cell treatment with PB extract

For assessing the PB extract's effect on MSCs' redox status, cells were seeded into 75 cm^2 flasks in DMEM containing 10% FBS. After 24 h incubation at 37°C in 5% CO_2, the cells were treated with different concentrations of PB extract in DMEM, 10% FBS and again incubated for 24 h. Then, cells were detached by trypsinization and used for the determination of lipid peroxidation, protein oxidation and reduced glutathione (GSH) levels.

2.1.5. Assessment of thiobarbituric acid reactive substances (TBARS), protein carbonyls (CARB) and GSH levels in MSCs

After treatment with PB extract, the cells were detached using trypsin, mixed with a PBS buffer and disrupted by vigorous vortexing. The protein concentration in the resulting cell lysates was determined using the Bradford assay. Next, a slightly modified TBARS assay as described previously [41] was employed. Specifically, for the assay, a total of (400-X) μl of PBS (where X represents the quantity of cell suspension required to obtain 30 μg of protein) or 400 μL of PBS for the blank was combined with 500 μl of Tris-HCl (200 mM, pH 7.4) and 500 μL of 35% TCA. The mixture was then incubated for 10 min at room temperature. Subsequently, a 1 mL solution consisting of 2 M Na_2SO_4 and 55 mM thiobarbituric acid was added and the samples were incubated at 95 °C for 45 min. After cooling the samples on ice for 5 min and vortexing, 1 mL of 70% TCA was added. The samples were then subjected to centrifugation at 15,000 x g for 3 min, and the absorbance of the resulting supernatant was measured at 530 nm. The concentration of TBARS was calculated based on the molar extinction coefficient of malondialdehyde and expressed as TBARS nmol per mg of protein in the cell lysate. Each experiment was repeated at least three times.

For the assessment of protein oxidation, CARB levels were assessed as described previously [42]. In brief, following the trypsinization process, the cellular suspension was homogenized by subjecting it to sonication while kept on ice. Then, 200 μL of 20% TCA was added to 200 μL of the cellular suspension, and this mixture was incubated in an ice bath for 15 min. Afterwards, it was centrifuged at 15,000 g for 5 min at 4°C. The resulting supernatant was discarded, and 500 μL of 2,4-Dinitrophenylhydrazine (DNPH) [in 2.5 N hydrochloride (HCl)] was added to the pellet for the sample, while 500 μL of 2.5 N HCl was added for the blank. The samples were then incubated in the dark for 1 hour, with intermittent vortexing every 15 min, and subsequently centrifuged at 15,000 g for 5 min at 4 °C. The supernatant was once again discarded, and 1 mL of 10% TCA was added to the samples, which were vortexed and centrifuged at 15,000 g for 5 min at 4 °C. After discarding the supernatant, 1 mL of ethanol-ethyl acetate (1:1 v/v) was added to the samples, which were vortexed and centrifuged at 15,000 g for 5 min at 4 °C. This washing step was repeated twice. The supernatant was discarded, and 1 mL of 5 M urea (pH 2.3) was added to the samples, which were then vortexed and incubated

at 37 °C for 15 min. Subsequently, the samples were centrifuged at 15,000 g for 3 min at 4 °C, and the absorbance was measured at 375 nm. It should be noted that the assay necessitates a minimum of 30 μg of protein in the tested sample. The total protein content in the cellular suspension was determined using the Bradford assay. The calculation of CARB concentration was based on the molar extinction coefficient of DNPH. Each experiment was repeated at least three times.

GSH levels were determined as described previously [41]. Following the trypsinization process, the cells were suspended in a PBS buffer and then lysed using sonication. Subsequently, the protein concentration in the cell lysates was determined using the Bradford assay. The next step involved performing the GSH assay, wherein a reaction mixture of 1 mL was prepared. This mixture consisted of 520 μL of 67 mM sodium phosphate buffer (pH = 8.0), 150 μl of cell lysate suspension containing 30 μg of protein, and 330 μL of 1 mM 5,5'-dithiobis(2-nitrobenzoic acid) (DTNB) solution. The samples were thoroughly mixed and incubated at room temperature in the dark for 15 min, while the absorbance was monitored at 412 nm. The concentration of GSH was then calculated based on the millimolar extinction coefficient of DTNB and expressed as nmol GSH per mg of protein in the cell lysate. Each experiment was repeated at least three times.

2.2. Study design and procedure

Individuals with diagnosis of T2DM, not insulin dependent, 30–70 years age, clinically and biochemically stable, without any acute metabolic complications of diabetes were also considered for the study. Statistical analyses were performed on the data of 10 subjects (5 males and 5 females) who completed the study. These numbers were chosen based on the literature where similar numbers had provided adequate power [26, 38, 43, 44]. An informed written consent was obtained from the participants. All procedures followed were conducted according to the guidelines laid down in the Declaration of Helsinki and following ethical approval provided by the Bioethics Committee of University of Thessaly, Volos (Approval no. 72/10.07.2023) and registration by the Council of University Hospital of Larissa (Approval no. 8882/28.02.2023).

The participants were selected by convenience purposive sampling from the diabetes patients who were under medical care the Endocrinology and Metabolic Diseases clinic of University Hospital of Larissa. Inclusion criteria were as follows: involvement in type 2 diabetes diagnosed by as FBG ≥ 126 mg/dl and glycated hemoglobin (HbA1c) ≥ 6.5% confirmed by endocrinologist diabetes, history of < 10 years, and use of metformin as the hypoglycemic agent. Exclusion criteria were: body mass index (BMI) ≥ 40, pregnancy, smoking, severe stress or pain, heart attack in the last 6 months, gastrointestinal disorders and surgeries during the last 6 months, liver or kidney failure, cancer, and taking steroids, antibiotics,

levothyroxine and other medications with an effect on glycemic control [45].

At the first week following the second screening, study participants in groups of four visited the Metabolic Unit of University Hospital of Larissa, where they were administered with a 50 g oral glucose load (reference test). An interview was performed to obtain information on the participants' demographics, diabetes duration, and history of diseases, medications, and the dose and the schedule of hypoglycemic agents. The participants were asked not change their diet and physical activity during the study. Additionally, they answered a 24-hour dietary recall for energy intake assessment and completed a Food Frequency Questionnaire (FFQ) [46], the International Physical Activity Questionnaire (IPAQ) [47] to evaluate their lifestyle habits. At the second and third week the study participants were provided with and consumed the two snacks respectively, each portion of which provided 25g of carbohydrates. When they consumed the PB snack-bar an organoleptic acceptance questionnaire [48] answered to evaluate color, texture, taste, overall liking and also their satiety assessed by using visual analogue scale (VAS), adopted from Flint et al [49]. Adverse effects, such as nausea, headache and flatulence, were also assessed (questionnaire) during the supervision period. Screening and enrollment of subjects started in November 2023, and all study procedure (Figure 1) were completed before May 2024.

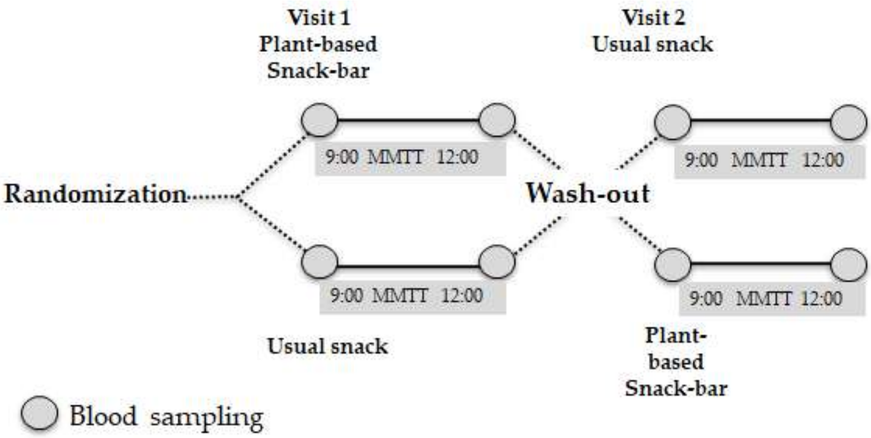

Figure 1. Study design and procedure: The participants underwent the mixed meal tolerance test on 2 separate days, 1 week apart. The participants were randomly assigned to two groups and had a plant-based, mushroom powder-fortified snack-bar (PBSB) or a usual snack. After 1 week, the participants were provided with the PBSB and the usual snack in the reverse order. MMTT: mixed meal tolerance test, blood samples: Glucose, Insulin etc.

2.2.2. Blood collection procedure

Subjects were asked to attend the Endocrinology and Metabolic Diseases clinic of University Hospital of Larissa over two days (each day with one type of test meal) which were 1 week apart, during morning hours (8:00 to 8:30 am). Prior to blood sampling and consumption of the test meals they remained fasted over 12

hours, were restricted to maximum 10 minutes of mild walking and were advised to refrain from taking their medications during the morning of each measurement. An intravenous cannula was placed in a forearm vein and blood samples were drawn at a fasting state (T0) and then at 30, 60, 90 and 120 min after the consumption of test meals. At each time point blood glucose and insulin levels were assessed.

2.2.3. Test meals

The current study examined the glycemic and insulinemic responses observed after consumption of two snacks, specifically wholegrain bread and low fat cheese (meal 1) and plant-based snack-bar using Mushroom (*Coprinus comatus*) powder (meal 2) (Figure 2). The test snacks were consumed by study participants between 8:30 and 9:00 a.m. and within 10–15 min under a researcher's supervision, following collection of the blood sample at T0. No parallel fluid intake was allowed. The portion size in the case of each one of the two test meals was adjusted to contain 25 g of carbohydrates. However, the two test snacks were considerably differentiating among them with respect to their content in calories and the remaining macronutrients and micronutrients. The caloric and nutrient content of the two test snacks is summarized in Table 1. The ingredients of the PB snack bar have been mentioned in previous publication (2023) [30].

Table 1. Nutritional profile of a plant-based snack-bar compared with the usual snack.

Nutrition declaration	**Snack 1 Study snack**	**Snack 1 (portion)**	**Snack 2 Study snack**	**Snack 2 (portion)**
Total energy, Kcal	240	160.9	266	101.4
Protein, g	13	10	11.8	4.5
Fat, g	6.8	4.4	12.6	4.8
Saturated, g	3.6	3.3	9.9	3.8
Carbohydrate, g	25	12.5	25	9.5
Fiber, g	3.6	1.8	2.5	0.96
Total sugars, g	3	1.5	2.94	1.12
Na, mg	0.93	0.5	37.8	14.4
K, mg	0	0	31.5	12
Foods consumed	Wholegrain bread, 60g and cheese (low fat), 25g	Wholegrain bread 30g and cheese (low fat), 25gr	PB Snack-bar 63gr	PB Snack-bar 24gr

*Snack 1/portion (en, %): vitamin B1 1%, vitamin B12 0%, calcium 28%, iron 2%, folate 6%,potassium 2%, magnesium 2%, phosphorus 23% and Snack 2/potion (en %): vitamin B12 20%, calcium 15%, iron 8%, folate 6%,potassium 10%, magnesium 25%, phosphorus 20%

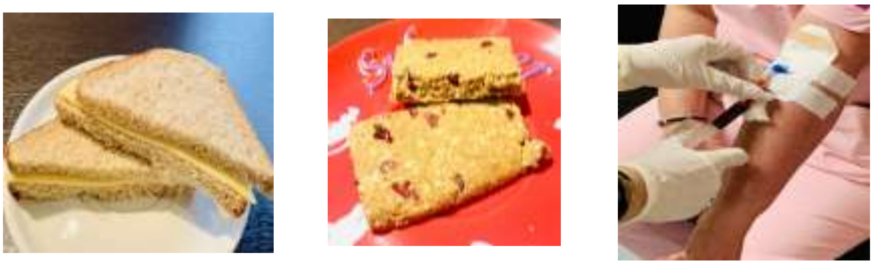

(**a**) (**b**) (**c**)

Figure 2. (**a**) Picture of the first snack of the study: 60g whole wholegrain bread and 25g low fat cheese; (**b**) Picture of the second snack of the study: 63g of PB snack-bar; (**c**) Blood collection procedure.

2.2.4. Anthropometric measurements

Anthropometry was carried out during the second screening as well as before blood collection at each time point of examination. Participants' weight was measured in light clothing and without shoes using a portable calibrated electronic weighing scale precision scale (TANITA MC-780U Multi Frequency Segmental Body Composition Analyzer, Amsterdam, the Netherlands). Height was measured with portable measuring inflexible bars (Seca model 220, Seca, Hamburg, Germany). Body weight and height were measured with study participants wearing light clothing and no shoes and all measurements were taken twice, and the average of the two values was reported and also body fat, visceral fat and muscle mass measurements took place. Body mass index (BMI) was calculated as weight (kg) divided by height squared (m^2). Waist (at umbilicus) and hip (at widest point) circumferences (WC and HC) were measured according to standard conditions using a measuring tape, and waist/hip circumference ratio (WHR) was calculated. Except from the anthropometric measurements, blood pressure was measured with a blood monitor (FYGB-869).

2.2.5. Biochemical indices

Blood was collected in vacutainers with no added anticoagulant and was kept at room temperature for approximately two hours, where it was allowed to clot as this was designated for serum separation. Centrifugation for serum separation was conducted at 3000 rpm for 15 min. Aliquots of 1.5 mL were then pipetted into plastic Eppendorf tubes and stored at −80 °C. Serum insulin was measured by an Electrochemiluminescence immunoassay (ECLIA) using commercially available kits (Roche Diagnostics GmbH D 68,298 Mannheim, Germany). Total cholesterol, HDL-C, triglycerides, glucose, alanine aminotransferase (ALT), aspartate aminotransferase (AST), alkaline phosphatase (ALP), γ glutamyl transferase (GGT), prothrombin time (PT) were measured by standard methods on an automatic analyzer (Olympus 600; Medicon, Athens, Greece). Specifically regarding plasma glucose, this was measured by an automated enzymatic assay (hexokinase) [50]. Additionally, ferritin, albumin, creatine, TSH, FT4, were measured using enzymatic colorimetric methods with commercially available

kits (COBAS 311, Roche Diagnostics GmbH, Mannheim, Germany). LDL-C was calculated according to the Friedwald equation. All the laboratory investigation took place at the Department of Endocrinology and Metabolic Diseases and HbA1c levels were estimated according the methodology that has been described to the previous study (2024) of the same team [3]. As concern trace elements levels were determined using wet acid digestion method that has been described by Wolide et al [51]. Levels of 25(OH)D_3 were measured by radioimmunoassay (RIA) in a two-step procedure as has been described by Kostoglou-Athanassiou et al [52]. Vitamin B12 estimation was done by a solid phase, competitive chemiluminescent enzyme immunoassay on Immulite 1000 analyzer using commercial kits from Siemens Healthcare Diagnostics Inc., (New York, USA) [53].
